# n-3 Polyunsaturated Fatty Acid Supplementation Has No Effect on Postprandial Triglyceride-Rich Lipoprotein Kinetics in Men with Type 2 Diabetes

**DOI:** 10.1155/2016/2909210

**Published:** 2016-02-29

**Authors:** André J. Tremblay, Benoît Lamarche, Jean-Charles Hogue, Patrick Couture

**Affiliations:** ^1^Institute of Nutrition and Functional Foods, Laval University, Quebec City, QC, Canada G1V 0A6; ^2^CHUQ Research Center, Laval University, Quebec City, QC, Canada G1V 4G2

## Abstract

Dietary n-3 polyunsaturated fatty acids (PUFAs) have been proposed to modulate plasma lipids, lipoprotein metabolism, and inflammatory state and to reduce triglyceride (TG) concentrations. The present double-blind, randomized, placebo-controlled, crossover study investigated the effects of n-3 PUFA supplementation at 3 g/d for 8 weeks on the intravascular kinetics of intestinally derived apolipoprotein (apo) B-48-containing lipoproteins in 10 men with type 2 diabetes.* In vivo* kinetics of the TG-rich lipoprotein (TRL) apoB-48 and VLDL apoB-100 were assessed using a primed-constant infusion of L-[5,5,5-D_3_] leucine for 12 hours in a fed state. Compared with the placebo, n-3 PUFA supplementation significantly reduced fasting TG concentrations by −9.7% (*P* = 0.05) but also significantly increased plasma levels of cholesterol (C) (+6.0%, *P* = 0.05), LDL-C (+12.2%, *P* = 0.04), and HDL-C (+8.4, *P* = 0.007). n-3 PUFA supplementation had no significant impact on postprandial TRL apoB-48 and VLDL apoB-100 levels or on the production or catabolic rates of these lipoproteins. These data indicate that 8-week supplementation with n-3 PUFAs in men with type 2 diabetes has no beneficial effect on TRL apoB-48 and VLDL apoB-100 levels or kinetics.

## 1. Introduction

The characteristic features of dyslipidemia that are associated with insulin-resistant states include elevated plasma triglycerides (TG) due to overaccumulation of TG-rich lipoproteins (TRL) of both intestinal (apoB-48) and hepatic origin (apoB-100), low HDL-cholesterol (C) levels, and the formation of small, dense LDL particles [1]. Several lines of evidence have suggested that apoB-48-containing particles are proatherogenic and are associated with increased cardiovascular disease risk [1]. In this regard, consumption of long-chain n-3 polyunsaturated fatty acids (PUFAs) is an efficacious approach to modify cardiovascular risk by improving dyslipidemia, hypertension, and endothelial function [2]. Supplementation with dietary n-3 PUFA has been consistently shown to reduce TG concentrations [3]. However, the mechanisms responsible for the hypotriglyceridemic effects of n-3 PUFAs have not been fully elucidated. The objective of the current study was to investigate the impact of n-3 PUFAs on the* in vivo* kinetics of intestinally derived apoB-48-containing lipoproteins and hepatic VLDL apoB-100 in men with type 2 diabetes. We hypothesized that n-3 PUFAs would beneficially affect plasma TRL levels by reducing the production and increasing the catabolism of both intestinal and hepatic lipoproteins.

## 2. Methods

### 2.1. Subjects

Ten men with type 2 diabetes as defined by the American Diabetes Association were recruited in Quebec City area to participate in the study. To be included in the study, participants had to have received stable doses of metformin for at least 3 months before randomization. All eligible subjects were required to discontinue their use of lipid-lowering medications for at least 6 weeks before blood sample collection. Subjects with monogenic lipid disorders; type 1 diabetes; insulin treatment; a previous history of cardiovascular disease; a recent history of alcohol or drug abuse; disorders of the hematologic, digestive, or central nervous system; known impairment of renal function; persistent elevations of serum transaminases; uncontrolled diabetes mellitus (HbA1c > 8.5%); or a history of cancer or of any other conditions that may interfere with optimal participation in the study were ineligible. The study was undertaken using a double-blind, randomized, crossover design with two balanced [1 : 1] treatments of 8 weeks each. All subjects received the following in a random order: 5 g/d (5 × 1 g capsules) of fish oil providing 3 g/d of EPA (64%) and DHA (36%) or a control supplementation (5 × 1 g capsules/d of corn and soybean oil). The treatments were separated by a 12-week wash-out period. During a 4-week run-in stabilization period that preceded the treatments, the participants were advised to consume a low-fat diet following the recommendations of the National Cholesterol Education Program, Adult Treatment Panel III. Dietary intake of marine-derived LCn-3PUFA was limited by prohibiting the consumption of fish throughout the entire experimental period, including during the wash-out period. The consumption of alcohol, vitamin supplements, and natural health products was also strictly forbidden throughout the entire experimental period. Fasting blood samples and kinetic studies using primed-constant infusion of deuterated leucine were performed following each phase of treatment. Compliance was assessed by capsule counting. The research protocol was approved by the Laval University Medical Center ethical review committee, and written informed consent was obtained from each subject. This trial was registered at clinicaltrials.gov as NCT01449773.

### 2.2. Characterization of Plasma Lipids and Lipoproteins

Twelve-hour fasting venous blood samples were obtained from the subjects' antecubital veins prior to the beginning of the kinetic study. Serum was separated from the blood cells by centrifugation at 2060 g for 10 minutes at 4°C. Plasma lipids and lipoproteins were measured as previously described [4].

### 2.3. Experimental Protocol for* In Vivo* Stable Isotope Kinetics

To determine the kinetics of TRL apoB-48 and VLDL apoB-100, the subjects underwent a primed-constant infusion of L-[5,5,5-D_3_] leucine while they were in a constantly fed state as previously described [4]. The kinetics of TRL apoB-48 and VLDL apoB-100 were derived by a multicompartmental model as previously described [4].

### 2.4. Statistical Analysis

Wilcoxon signed-rank tests were used to compare the effects of n-3 PUFA on the fasting lipid-lipoprotein profile and kinetic parameters. Differences were considered significant at *P* ≤ 0.05. All analyses were performed using JMP Pro Statistical Software (version 12, SAS Institute, Cary, NC).

## 3. Results

### 3.1. Demographic Characteristics and Fasting Biochemical Parameters of Subjects

Table 1 shows the demographic characteristics and fasting biochemical parameters of the 10 patients with type 2 diabetes following an 8-week supplementation with either placebo or n-3 PUFAs (3 g/d). The mean age of the participants was 54.7 ± 7.6 years. No significant differences were observed in body weight, body mass index, or systolic blood pressure between the two supplementation phases. Supplementation with n-3 PUFA significantly reduced plasma TG concentrations by −9.7% (*P* = 0.05) but significantly increased levels of plasma cholesterol (+6.0%, *P* = 0.05), LDL-C (+12.2%, *P* = 0.04), and HDL-C (+8.4%, *P* = 0.007). No significant differences in fasting plasma levels of apoB or apoAI were observed between the two treatments. Compared with the placebo, n-3 PUFA supplementation had no significant impact on glucose or insulin concentrations or HbA1C levels. As shown in Table 2, n-3 PUFA supplementation had no significant effect on TRL apoB-48 or VLDL apoB-100 kinetics in men with type 2 diabetes.

## 4. Discussion

In the present study, supplementation with n-3 PUFAs at a dose of 3 g/d for 8 weeks significantly reduced fasting plasma TG levels but increased plasma cholesterol, LDL-C, and HDL-C concentrations in men with type 2 diabetes compared with a placebo. In addition, n-3 PUFA supplementation had no significant effect on the postprandial secretion and clearance of TRL apoB-48 or VLDL apoB-100 in these subjects.

It is well documented that type 2 diabetes is associated with hypertriglyceridemia and elevated levels of apoB-100- and apoB-48-containing lipoproteins [4]. n-3 PUFAs have long been used in the treatment of hypertriglyceridemia, as they reduce the hepatic production of VLDL, favor fatty acid oxidation, and enhance VLDL clearance [5]. As expected, n-3 PUFA supplementation for 8 weeks significantly reduced fasting TG levels, which is in agreement with previous observations from other groups [6, 7]. Our results also showed that n-3 PUFA supplementation increased total and LDL-C concentrations in a manner consistent with previous findings [8–10].

Although n-3 PUFA supplementation significantly reduced TG levels in a fasting state, postprandial concentrations of VLDL apoB-100 were not significantly different after the two interventions. Our results showed no significant changes in VLDL apoB-100 fractional catabolic and production rates, a finding that contrasts with previous kinetic studies. Nestel et al. [11] reported that dietary n-3 PUFAs lowered secretion rates of both VLDL apoB and VLDL TG in the absence of changes in the clearance of VLDL. In type 2 diabetic patients, n-3 PUFAs were effective at lowering TG concentrations via the suppression of VLDL production and the stimulation of its conversion into LDL, with no change in the apoB-100-containing lipoprotein clearance rate [12]. Earlier studies on VLDL TG kinetics on subjects who were fed between 10 and 17 g/d of fish oil reported a significant reduction in VLDL TG synthetic rate and an increased clearance rate [13]. In cultured rat hepatocytes, EPA and DHA inhibited VLDL apoB secretion by 31% and 54%, respectively [14]. Moreover, a study in African green monkeys showed that perfused monkey livers had decreased secretion of cholesteryl ester and TG but maintained the same apoB output, suggesting that production of the same number of smaller, TG-depleted apoB particles likely compensated for the decreased availability of TG for VLDL secretion [15].

The impact of n-3 PUFA supplementation on apoB-48 metabolism has been recently investigated. Levy et al. [16] showed that the jejunal secretion of apoB-48 from diabetic and insulin-resistant rats following dietary intake with n-3 PUFAs was significantly reduced, and this was most likely the result of posttranslational degradation. Moreover, apoB mRNA and corresponding apoB secretion were reduced in intestinal/colonic-derived Caco-2 cultured cells when incubated with EPA [16]. In JCR:LA-cp rats, n-3 PUFA treatment resulted in a significant improvement in apoB-48 and TG postprandial response compared with controls [17]. In healthy subjects, n-3 PUFA supplementation (4 g/d) for 4 weeks reduced postprandial TG, apoB-48, and apoB-100 concentrations [5]. However, this is in contrast with our results showing no significant effect of n-3 PUFA supplementation on apoB-48 production and clearance rates.

This study has several strengths. The relatively large number of participants and the robust study design (double-blind, randomized, crossover) increased our statistical power. Our specific inclusion criteria also limit the variability related to the background care provided. The statistical analyses were undertaken in a blinded fashion and according to the a priori defined plan and hypothesis testing. Our study also shows that n-3 PUFA supplementation had no significant impact of postprandial TRL apoB-48 kinetics but significantly modulated both fasting TG and LDL-C concentrations. Several potential mechanisms could explain these observations. (1) The meals provided during the kinetic study did not contain n-3 PUFAs, and this could have influenced postprandial response and attenuated n-3 PUFA effects on VLDL apoB-100 and TRL apoB-48 secretion. Recent studies have suggested that the effect of the meal may in fact overwhelm the lipid-lowering effect of the n-3 PUFA supplementation [18]. (2) It is also likely that the modest hypertriglyceridemia could have attenuated the beneficial impact of n-3 PUFA supplementation. Across studies, higher baseline TG levels were associated with greater reductions in serum TG levels following n-3 PUFA supplementation [19]. (3) In addition, we cannot rule out the possibility of longer term effects of n-3 PUFA supplementation on TRL metabolism. (4) Finally, it is likely that higher daily dosages of n-3 PUFAs (>3 g/d) would have led to greater differences in apoB kinetics in subjects with type 2 diabetes. Previous studies have shown a strong correlation between daily dosages of n-3 PUFAs and the reductions in serum TG levels [19].

## 5. Conclusion

n-3 PUFA supplementation for 8 weeks was effective at reducing fasting TG levels in subjects with type 2 diabetes but also increased levels of plasma cholesterol, LDL-C, and HDL-C. However, no significant effect was observed on postprandial VLDL apoB-100 and TRL apoB-48 levels or kinetics.

## Figures and Tables

**Table 1:** Characteristics and fasting lipid/lipoprotein profile of the evaluated patients with type 2 diabetes.

	Placebo (*n* = 10)	n-3 PUFA (*n* = 10)	%Δ	*P*
Body weight, kg	106.1 ± 19.5	105.8 ± 21.0	−0.3	0.7
Body mass index, kg/m^2^	34.1 ± 5.5	34.1 ± 6.1	—	0.7
Serum				
Cholesterol, mmol/L	4.68 ± 0.80	4.96 ± 0.58	+6.0	0.05
Triglycerides, mmol/L	2.58 ± 1.12	2.33 ± 0.94	−9.7	0.05
LDL-cholesterol, mmol/L	2.55 ± 0.72	2.86 ± 0.47	+12.2	0.04
HDL-cholesterol, mmol/L	0.95 ± 0.17	1.03 ± 0.20	+8.4	0.007
Apolipoprotein B, g/L	1.00 ± 0.19	1.03 ± 0.11	+3.0	0.4
Apolipoprotein AI, g/L	1.12 ± 0.18	1.15 ± 0.14	+2.6	0.4
Glucose homeostasis				
Glucose, mmol/L	7.3 ± 1.6	8.1 ± 2.2	+11.0	0.3
Insulin, *ρ*mol/L	147 ± 86	164 ± 109	+11.9	0.6
HbA1c	0.070 ± 0.010	0.072 ± 0.011	+2.9	0.1

PUFA: polyunsaturated fatty acid.

Mean ± SD; % represents the percentage of difference between the two intervention phases.

**Table 2:** Kinetic parameters of apoB-48 in TRL and apoB-100 in VLDL following supplementation with n-3 PUFA in patients with type 2 diabetes.

	Placebo (*n* = 10)	n-3 PUFA (*n* = 10)	%Δ	*P*
TRL apoB-48				
Pool size, mg	99 ± 62	96 ± 62	−2.6	0.9
Fractional catabolic rate, pools/day	6.9 ± 3.8	7.6 ± 2.8	+10.0	0.3
Production rate, mg/kg/day	5.4 ± 3.2	6.0 ± 3.8	+11.1	0.7
VLDL apoB-100				
Pool size, mg	600 ± 258	594 ± 321	−0.9	0.9
Fractional catabolic rate, pools/day	6.9 ± 2.3	6.9 ± 2.4	—	0.9
Production rate, mg/kg/day	35.6 ± 12.6	33.9 ± 12.4	−5.6	0.6

PUFA: polyunsaturated fatty acid.

TRL: triglyceride-rich lipoproteins.

Mean ± SD; %Δ represents the percentage of difference between the two intervention phases.

## Abbreviations

Apo: Apolipoprotein
DHA: Docosahexaenoic acid
EPA: Eicosapentaenoic acid
HbA1c: Glycated hemoglobin
HDL: High-density lipoprotein
LDL: Low-density lipoprotein
PUFA: Polyunsaturated fatty acid
TRL: Triglyceride-rich lipoprotein
VLDL: Very-low-density lipoprotein.
